# Guided Wave Propagation in Detection of Partial Circumferential Debonding in Concrete Structures

**DOI:** 10.3390/s19092199

**Published:** 2019-05-13

**Authors:** Beata Zima

**Affiliations:** Department of Mechanics of Materials and Structures, Faculty of Civil and Environmental Engineering, Gdańsk University of Technology, Narutowicza 11/12, 80-233 Gdansk, Poland; beazima@pg.edu.pl

**Keywords:** debonding, damage detection, piezoelectric transducer, guided waves, reinforced concrete, nondestructive testing

## Abstract

The following article presents results of investigating the damage detection in reinforced concrete beams with artificially introduced debonding between the rod and cover, using a non-destructive method based on elastic waves propagation. The primary aim of the research was to analyze the possible use of guided waves in partial circumferential debonding detection. Guided waves were excited and registered in reinforced concrete specimens with varying extents of debonding damage by piezoelectric sensors attached at both ends of the beams. Experimental results in the form of time–domain signals registered for variable extent of debonding were compared, and the relationships relating to the damage size and time of flight and average wave velocity were proposed. The experimental results were compared with theoretical predictions based on dispersion curves traced for the free rod of circular cross-section and rectangular reinforced concrete cross-section. The high agreement of theoretical and experimental data proved that the proposed method, taking advantage of average wave velocity, can be efficiently used for assessing debonding size in reinforced concrete structures. It was shown that the development of damage size in circumferential direction has a completely different impact on wave velocity than development of debonding length. The article contains a continuation of work previously conducted on the detection of delamination in concrete structures. The proposed relationship is the next essential step for developing a diagnostics method for detecting debondings of any size and orientation.

## 1. Introduction

Debonding is one of the most frequent and most dangerous damages to structures made of different materials. Its occurrence leads to the loss of properties that result from the cooperation of various materials, and significantly reduces the load capacity of the entire structure. In addition, it usually develops inside the structure, which precludes detection during standard visual inspection. For this reason diagnostic methods should be used to detect internal damages without interfering with the integrity or disassembly of tested object [1,2,3,4].

A very promising non-destructive approach is the guided wave propagation method. Long-range diagnostic capability of elastic waves makes them one of the most attractive tools for non-destructive evaluation (NDE) and structural health monitoring (SHM) systems. Their ability to travel long distances without significant amplitude loss [5], even in specimens of complex shapes and geometries, has been used for detecting and quantifying damages in various types of structures. The idea of wave application in diagnostics involves placing an actuator, which excites wave motion in investigated specimen. The excited wave is captured by sensors as time–domain signals, which is then properly interpreted. Because waves travel in all directions, and transducers locations can be freely chosen, guided waves allow for monitoring those parts of the structure, which normally remain unavailable. Therefore, they have been effectively used in debonding detection in a variety of engineering objects. Li and Zhou [6] demonstrated an application of air-coupled ultrasonic testing in debonding detection in honeycomb sandwich composites. A possible application of guided waves in debonding detection in honeycomb sandwich composites was also described by Sikdar and Ostachowicz [7], who proposed an algorithm based on differential changes in mode amplitudes. Hidden debondings in multi-layer composites have been detected using Lamb waves by Sohn et al. [8], who constructed wavefield images to study the interaction of waves with delaminations. The detection of interfacial delamination in composite laminates using guided waves was investigated by Li et al. [9]. They showed that the time of flight of symmetric and antisymmetric fundamental modes changes as a result of delamination occurrence and proposed a method of evaluating the damage size based on those changes. Shoja et al. [10] showed the results of numerical simulations concerning guided wave propagation in composite laminates with delamination introduced into the model by local stiffness reduction. The problem of propagation of fundamental antisymmetric mode A_0_ in composite laminates with semi-infinite debonding was a topic of interest for Ramadas et al. [11]. They studied quantitatively the reflection and transmission of Lamb waves at the edge of the debonding in three different types of laminates (quasi-isotropic, unidirectional, and cross-ply). The time–domain spectral element method was employed by Munian et al. [12] to detect delamination in composites and to analyze the interaction of elastic waves with the damage. They studied the sensitivity of delamination to applied wavelengths. Studies concerning the interaction of Lamb waves with delamination edges in composites were carried out by Feng et al. [13]. They proposed a method for locating delamination using time of flight of symmetric and antisymmetric modes. In the paper [14] they also detected delamination in CFRP (carbon fiber reinforced polymer) using chirp-excited Lamb wave and wavelet analysis. Their detection method was based on assumption that for a specific length of delamination, only the particular frequency components are disturbed. Yelve et al. [15] used the Lamb wave-based nonlinear method in delamination detection in composites. They observed higher harmonics excited resulting from the contact nonlinearity at the damage and proposed a new hybrid debonding detection method based on spectral and temporal data. The sensitivity of scattering resonance frequencies to damage size and depth was used by Eremin et al. [16] in identifying delamination. The resonance frequency dependencies on damage depth and length obtained by the boundary integral equation method and finite element method have been verified experimentally for specimens with varying damage size.

Debonding occurrence is a particularly frequent damage of reinforced concrete structures. Because reinforced concrete is one of the most popular materials used in civil engineering, development of non-destructive methods for deboning detection has been of high priority in recent years. Debondings between steel bar and concrete cover varying in length were detected using guided waves by Na et al. [17]. They used a special coupler between rebar and transducer to induce propagation of high and low frequencies. Studies in References [18] and [19], by Wu and Chang, present results of theoretical, numerical, and experimental research concerning debonding detection in reinforced concrete beams. The authors show that debonding length influences signal amplitude, however, they did not register changes in the travel time of wave signals for varying debonding lengths. Continuous monitoring systems for detecting debonding between carbon fiber reinforced plastic and reinforced concrete structure was proposed by Kim et al. [20]. They showed that time reversal acoustic method can be applied for detecting the initiation and the region of debonding. Wang et al. [21] developed a concrete-steel spectral element to model guided wave propagation along rebar embedded in concrete. They performed the parametric studies to evaluate the influence of different damage scenarios on wave propagation phenomenon, which were then verified experimentally. Song et al. [22] proposed a damage index based on wavelet analysis, which allow the detection of cracks inside the concrete structure. Their methods was then extended by Xu et al. [23] to debonding detection in concrete-filled steel tube. Li et al. [24] reported the results of applying the time reversal technique in debonding detection in concrete beams under different CFRP bonding conditions. To assess the severity of damage, they proposed an indicative parameter, which was a correlation coefficient between the input signal and the extracted wave package from the reconstructed signal. The influence of debonding position on the complexity of wave propagation phenomenon was described by Zima and Rucka [25,26]. They investigated theoretically, numerically, and experimentally wave propagation in rods embedded in mortar block with circular cross-section and showed that debonding length and position have significant impact on wave amplitude and velocity. The influence of debonding length on wave amplitude was also shown in [27]. Zima and Kędra proposed a reference-free method for determining debonding length in reinforced concrete specimens based on monitoring time of flight of registered signals [28]. Chen et al. [29] showed that surface wave can be efficiently used in interface debonding of concrete-filled steel tubular detection. Nonlinear Rayleigh waves were utilized for debonding detection in CFRP-retrofitted concrete by Ng et al. [30].

**Figure 1:**
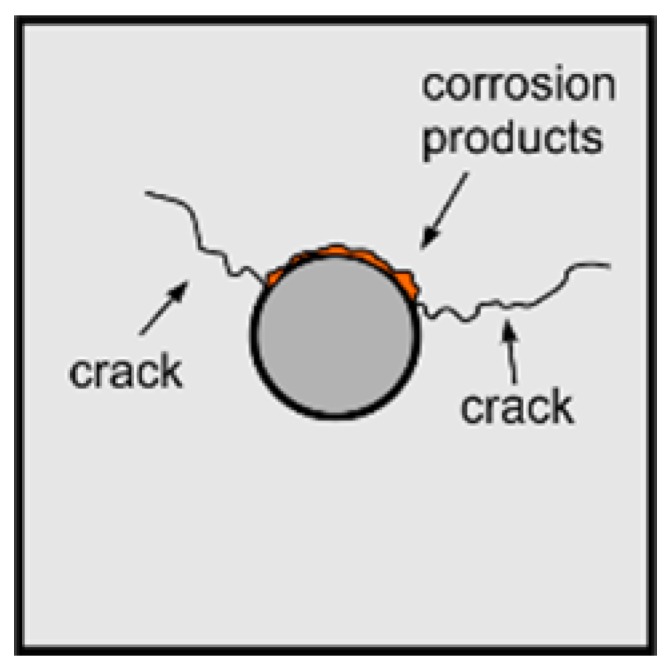
Partial circumferential debonding caused by corrosion damage in reinforced concrete beam.

Despite the fact that deboning detection in reinforced concrete elements experienced significant interest over the last decades, guided wave propagation methods still require greater accuracy to be viable tools for monitoring real structures. The influence of debonding length on wave propagation has been well described in papers [25,26]. However, in the majority of cases mentioned, only the length of debonding was variable. The circumferential extent remained unchanged and the rod was usually debonded around the whole circumference. The main unsolved issue is how partial circumferential debonding affects basic parameters of wave motion. Meanwhile, partial debonding detection is an especially important issue, because this type of damage can develop gradually (Figure 1). Partial circumferential debonding can be, i.e., a result of corrosion process: corrosion products tend to cause expansion, which in consequence apply a pressure to the surrounding cover. At the first stage of damage development, rod does not cooperate with concrete cover only in a certain area. An early damage detection significantly increases the chance of avoiding large repair costs or even replacing the entire structural elements.

This paper presents the results of the theoretical, numerical, and experimental investigation of wave propagation in reinforced concrete beams with partial circumferential debonding between steel rod and concrete cover. A number of concrete beams with the same length of debonding but varying extents of circumferential direction were investigated. Guided waves were excited and measured by piezoelectric sensors attached at both ends of the beams. The study discusses observed changes in time–domain signals collected for varying debonding size and varying excitation frequency. The main novel element is the approximate relationship between the average wave velocity in reinforced concrete beam and circumferential damage extent, which was proposed on the basis of experimental results. The obtained results prove that average wave velocity or time of flight can be effectively used as indicative parameters in monitoring circumferential debonding development in reinforced concrete structures. The main advantage of the presented approach is possibility its application without collecting any baseline data. The article contains a continuation of work previously conducted on the detection of delamination in concrete structures. The proposed relationship is an indispensable prerequisite for developing a diagnostics method based on guided waves, which would allow the detection of debondings of any size and orientation, then estimate the size of steel–concrete contact zone and in consequence determine the load capacity of the structure. It has been shown that damage development in circumferential direction has a completely different impact on wave velocity than development in longitudinal direction given in [28].

## 2. Theoretical Investigation of Wave Propagation in Concrete Beam

### 2.1. Theoretical Model

**Figure 2:**
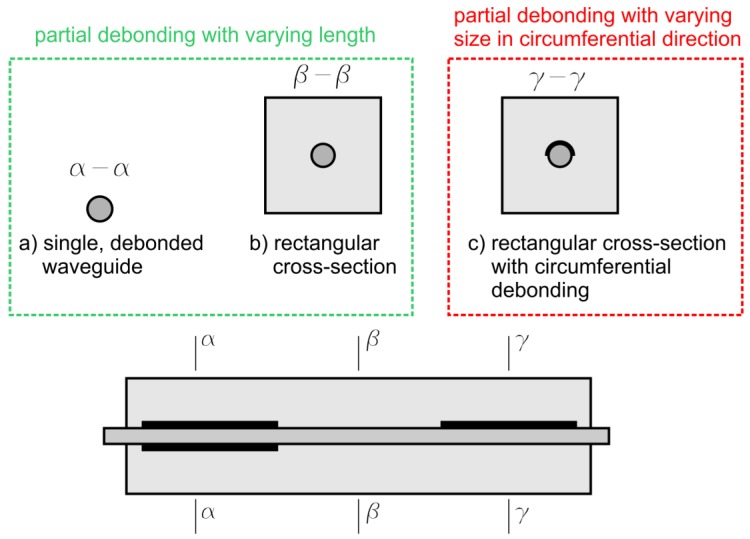
Types of cross-sections distinguished in partially debonded reinforced concrete beam: (**a**) single, debonded waveguide, (**b**) rectangular reinforced concrete cross-section, and (**c**) rectangular cross-section with circumferential debonding between concrete cast and steel rod.

Because guided waves are dispersive waves and their propagation velocity depends on excitation frequency, the description of wave propagation phenomenon requires consideration of the dispersion equation. Dispersion equations relate basic propagation parameters like group and phase propagation velocity or wavenumber and excitation frequency. The type of dispersion equation, which needs to be solved depends on specimen cross-section. Thus, when the wave is propagated in a complex specimen like reinforced concrete beam with partially debonded rod (see Figure 2), the first step is to determine, what types of cross-sections can be distinguished. Two extreme cases are presented in Figure 2. The first case concerns undamaged beam with perfect bonding between steel rod and concrete cover. The second extreme case is totally debonded rod, which can be then considered as single waveguide of circular cross-section. When circumferentially oriented debonding develops in a beam, a wide range of intermediate cases of rod partially bonded with the cover is possible. The case of partial debonding with varying length is well recognized (Figure 2a,b). The influence of circumferentially oriented debonding on wave propagation has not been considered yet (Figure 2c).

### 2.2. Dispersion Curves

In the first step, dispersion equations for debonded, free rod are considered. In the case of simple cross-sections, e.g., axisymmetric rods or plates made of elastic and isotropic material, equations are given explicitly. Propagation of longitudinal modes in rods of circular cross-section is described by Pochhammer–Chree equation [31,32]:

(1) $$\frac{2\alpha }{r}\left(\beta^{2}+k^{2}\right)J_{1}\left(\alpha r\right)J_{1}\left(\beta r\right)-{\left(\beta^{2}-k^{2}\right)}^{2}J_{0}\left(\alpha r\right)J_{1}\left(\beta r\right)-4k^{2}\alpha \beta J_{1}\left(\alpha r\right)J_{0}\left(\beta r\right)=0,$$

where *J*_1_ and *J*_0_ are Bessel’s functions of the first kind, *r* is a radius of the rod and parameters *α* and *β* are defined as follows:

(2) $$\alpha^{2}=\frac{\omega^{2}}{c_{P}^{2}}-k^{2},$$

(3) $$\beta^{2}=\frac{\omega^{2}}{c_{S}^{2}}-k^{2}.$$

pressure wave velocity and shear wave velocity denoted as *c_p_* and *c_s_*, respectively can be calculated using the formulas:

(4) $$c_{P}=\sqrt{\frac{E\left(1-\nu \right)}{\rho \left(1+\nu \right)\left(1-2\nu \right)}},$$

(5) $$c_{S}=\sqrt{\frac{E}{2\rho \left(1+\nu \right)}}.$$

The dispersion equation for flexural waves can be found in [33]:

(6) $$J_{1}\left(\tilde{\alpha }\right)J_{1}^{2}\left(\tilde{\beta }\right)\left[f_{1}J_{\tilde{\beta }}^{2}+f_{2}J_{\tilde{\alpha }}J_{\tilde{\beta }}+f_{3}J_{\tilde{\beta }}+f_{4}J_{\tilde{\alpha }}+f_{5}\right]=0.$$

where:

(7) $$\begin{array}{l} f_{1}=2{\left({\tilde{\beta }}^{2}-{\tilde{k}}^{2}\right)}^{2},\\ f_{2}=2{\tilde{\beta }}^{2}\left(5{\tilde{k}}^{2}+{\tilde{\beta }}^{2}\right),\\ f_{3}={\tilde{\beta }}^{6}-10{\tilde{\beta }}^{4}-2{\tilde{\beta }}^{4}{\tilde{k}}^{2}+2{\tilde{\beta }}^{2}{\tilde{k}}^{2}+{\tilde{\beta }}^{2}{\tilde{k}}^{4}-4{\tilde{k}}^{4},\\ f_{4}=2{\tilde{\beta }}^{2}\left(2{\tilde{\beta }}^{2}{\tilde{k}}^{2}-{\tilde{\beta }}^{2}-9{\tilde{k}}^{2}\right),\\ f_{5}={\tilde{\beta }}^{2}\left(-{\tilde{\beta }}^{4}+8{\tilde{\beta }}^{2}-2{\tilde{\beta }}^{2}{\tilde{k}}^{2}+8{\tilde{k}}^{2}-{\tilde{k}}^{4}\right), \end{array}.$$

and $\tilde{\alpha }$, $\tilde{\beta }$, $\tilde{k}$ are defined as dimensionless wavenumbers:

(8) $$\tilde{\alpha }=\alpha r,$$

(9) $$\tilde{\beta }=\beta r,$$

(10) $$\tilde{k}=kr.$$

function $J_{x}$ occurring in Equation (6) is Onoe’s function of the first kind and order unity [33]:

(11) $$J_{x}=J_{1}(x)=x\frac{J_{0}(x)}{J_{1}(x)}.$$

**Figure 3:**
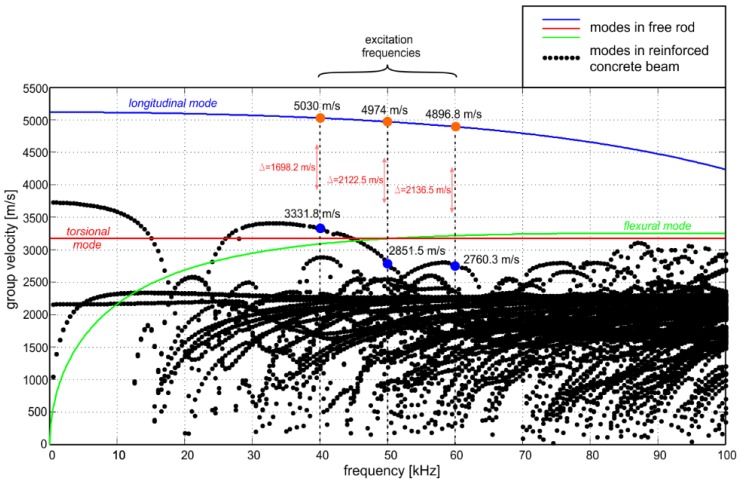
Group velocity dispersion curves of 2 cm diameter steel bar (E = 207 GPa, v = 0.3, and ρ = 7894 kg/m^3^) obtained analytically (solid lines) and dispersion curves for rectangular reinforced concrete cross-section (steel: E = 207 GPa, v = 0.3, ρ = 7894 kg/m^3^, concrete: E = 29 GPa, v = 0.2, and ρ = 2306 kg/m^3^).

Equations (1) and (6) were solved in MATLAB environment using free software PCDISP [34] and the solution in the form of dispersion curves plotted by solid lines is given in Figure 3. The calculations were performed for steel rod with circular cross-section and diameter of 2 cm. For the analyzed frequency range 0–100 kHz only one longitudinal (blue line) and one flexural mode (green line) can be excited.

In the case of more complex cross-sections dispersion equations cannot be formulated explicitly. The solution can be then obtained using numerical methods based on finite elements, e.g., [35,36,37]. Finite elements methods allow for obtaining dispersion solution for any cross-section, however, when calculations need to be performed repeatedly, they may be associated with high computational costs.

The rectangular concrete cross-section with the steel core given in Figure 2b requires the use of the finite elements approach. The dispersion curves for analyzed cross-section were obtained using the Graphical User Interface for Guided Ultrasonic Waves (GUIGUW) program based on semi-analytical finite element method [38]. The GUIGUW allows tracing the curves for all mode families (longitudinal, flexural, and torsional), however, the mode type can be recognized only by identifying the associated wave structures.

The obtained results suggest a huge discrepancy between wave propagation phenomenon observed for single waveguide and waveguide embedded in concrete block. The first difference is the number of dispersion solutions, which increases significantly with the complexity of the investigated cross-section. For a single waveguide, only one longitudinal, one flexural, and one torsional can be excited, while the number of possible wave modes for reinforced concrete beam significantly hinders correct interpretation of dispersion solution (Figure 3). Unfortunately, when multiple modes can be propagated in specimen, registered signals are usually more difficult to interpret [26].

The second difference is the curves’ shape, and in consequence, wave velocity. The longitudinal mode in free rod is characterized by much higher velocity than any mode in the reinforced concrete beam. It can then be expected that for the analyzed frequency range 0–100 kHz and longitudinal excitation wave velocity would increase with the rod detachment from the concrete cover. The difference between velocities of particular modes has been already used in assessing total debonding length in concrete beams [28].

Flexural mode in free rod propagates faster than waves in concrete beam, but only above certain frequency (about 45 kHz). The torsional mode in both cases is characterized by constant velocity, regardless of the excitation frequency.

## 3. Experimental Investigation of Wave Propagation in Beams with Debonding

### 3.1. Experimental Model

**Figure 4:**
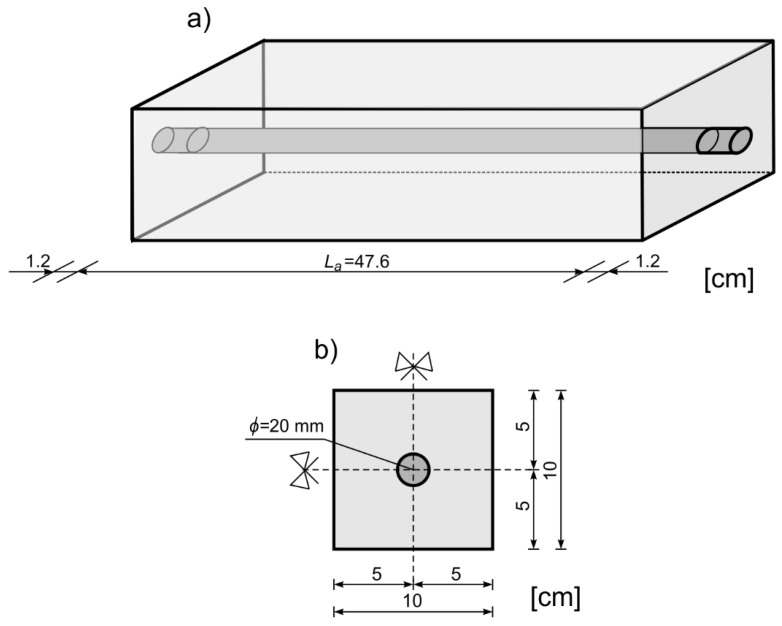
Experimental specimens**:** (**a**) geometry and (**b**) cross-section of the beam.

Experimental investigations were conducted on reinforced concrete beams with a rectangular cross-section. The beams consisted of one steel circular bar, with a diameter of *d_r_* = 2 cm and a length of 50 cm, embedded in the longitudinal direction in a concrete block. The dimensions of the beam were 10 cm × 10 cm × 47.8 cm. The beam geometry and cross-section are presented in Figure 4. Material parameters of the concrete were determined during destructive tests. The concrete samples with dimensions 150 mm × 150 mm × 150 mm were placed in hydraulically actuated and manually controlled testing machine. The measurement of strains was made using extensometer and strain gauge attached to the concrete sample. The material parameters obtained for concrete were as follows: *E* = 29 GPa, *v* = 0.2, and ρ = 2306 kg/m^3^. The concrete mixture was made of Portland cement type CEM I 42.R, with sand (0–2 mm), and fine aggregates (2–8 mm). The weight ratio of the sand and fine aggregates was 2:3. The material parameters of the steel were determined during tensile tests by extensometric measurement. The material parameters for steel were E = 207 GPa, *v* = 0.3, and ρ = 7894 kg/m^3^. All beams were left to cure in order to obtain full strength concrete (26 MPa) before the non-destructive experimental tests were conducted.

**Figure 5:**
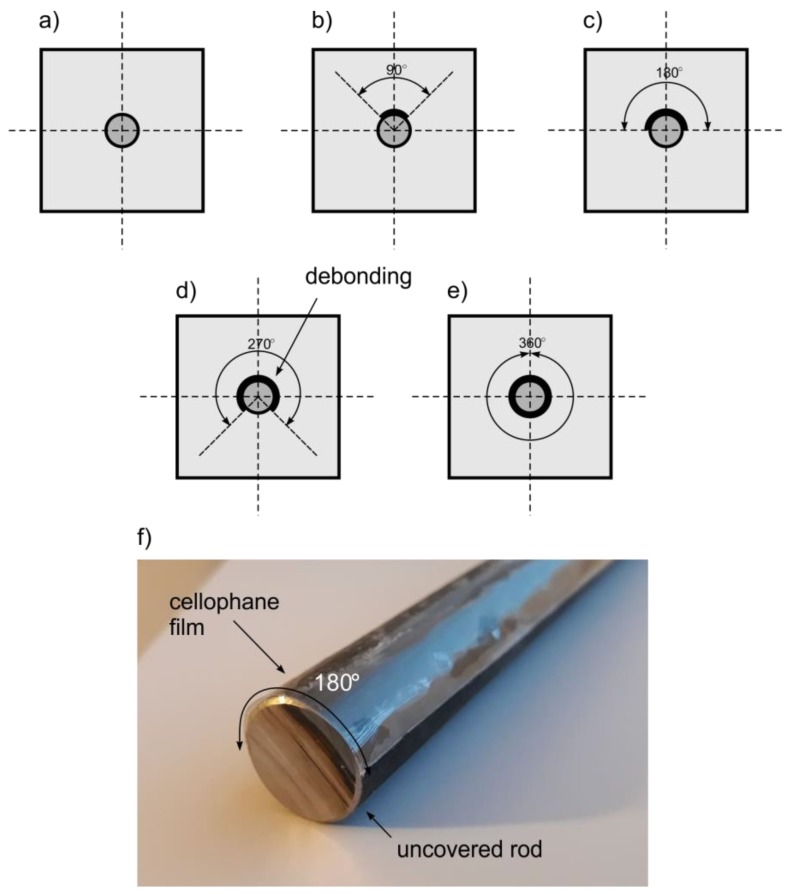
Schemes of investigated specimens: (**a**) beam #A with healthy bonded rod and beams with circumferential debonding with extent of (**b**) #B = 90, (**c**) #C = 180, (**d**) #D = 270, (**e**) #E = 360, and (**f**) a photograph of the rod wrapped in cellophane film.

A number of damage scenarios were investigated (Figure 5). In the first case the beam contained only one fully covered healthy rod (beam #A). In the other analyzed cases, rod was partially debonded in circumferential direction. The extent of the damage in beams #B–#E was equal to 90, 180, 270, and 360 degrees, respectively.

The rods were debonded along the whole length, thus, the total debonding length was the same in each case and was equal to 47.6 cm. Debonding was performed artificially by introducing cellophane film with a thickness of 90 µm, which is a very small size compared to the wavelength applied in experiment. For this reason, the damage of a comparable in size to the thickness of the cellophane film would not be easily detected. However, the cellophane film reduced an adhesion connection between rod and concrete, which provided lack of continuity of stresses and displacements at the border of media. Continuity of stresses and displacements fields is a basic assumption of wave propagation model in multilayered specimen [39]. When this assumption is not met, dispersion solutions given in Figure 3 for beam with perfectly bonded rod are not valid any more. Hence, wave propagation velocity in damaged beam should be described by different dispersion curves than velocity in undamaged beams.

### 3.2. Experimental Set Up

**Figure 6:**
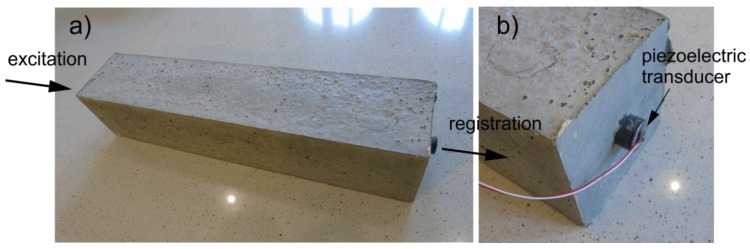
Experimental set up: (**a**) tested reinforced concrete beam and (**b**) a detail of transducer attached to rod embedded in beam.

In the prepared beams guided waves were excited and measured with the use of piezoelectric transducers Noliac NAC2012 attached at the both ends of the specimens. The dimensions of the transducers were 3 mm × 3 mm × 2 mm. The maximum operating voltage of the transcoders was 100 V. A free stroke for NAC2012 was 3.3 μm and a blocking force was 378 N. Waves were propagated and captured by PAQ-16000D signal generator. The amplitude of the input signal was equal to 10 V. The sampling rate was 2 MHz. The excitation signal was a wave packet comprising a ten-cycle sine function with a carrier frequency of 40, 50, or 60 kHz modulated by a Hanning window. The experimental model of the beam and a detail of transducer attached at the end are shown in Figure 6. When choosing the excitation frequencies, a number of different criteria, among others, the amplitude and readability of the signals and time duration of the excitation wave packets were taken into account. However, the main reason for choosing 40, 50, and 60 kHz, was the difference between velocities of the fastest modes in a free rod and reinforced concrete cross section determined on the basis of dispersion curves (see Figure 3). The excitation frequencies differed slightly and the difference between time duration of input wave packets was not considerable, while the difference in velocities of the fastest modes and in consequence, the sensitivity to debonding detection discussed in the further part of the paper was clearly visible.

### 3.3. Results of Experimental Investigation

**Figure 7:**
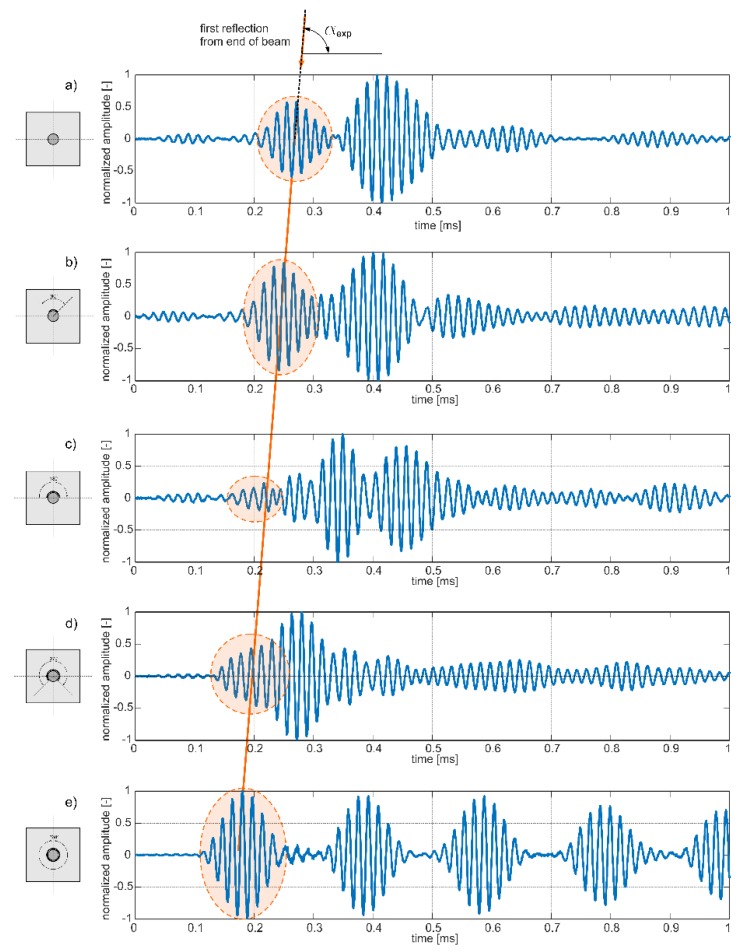
Experimental wave propagation signals registered at the end of the beam with variable circumferential debonding extent: (**a**) undamaged beam and beam with debonding extent of (**b**) 90°, (**c**) 180°, (**d**) 270°, and (**e**) 360° for excitation frequency 60 kHz.

Results of experimental investigations are presented in Figure 7 in the form of time–domain signals received at the end of the beam. The signals were normalized, so the maximum value was equal to 1. In each signal, the first reflection from the end of specimen was identified and highlighted. 

**Figure 8:**
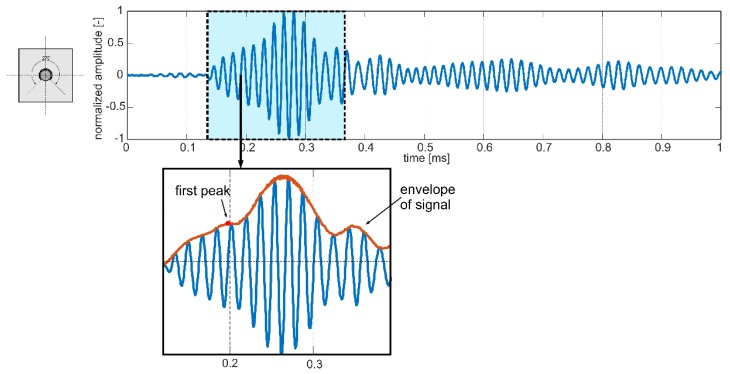
Initial part of the signal registered for the beam #D: determining the time of flight for overlapping wave packets.

Low-amplitude wave packets registered at the initial period of signals variability were recognized as avalanche breakdown and they should be omitted in the signal analysis process. In the case of the signal presented in Figure 7d, only the first part of the wave packet is highlighted. In this case, two different wave packets overlapped with each other and the time of flight only of the first disturbance was taken into account in the further investigations. In Figure 8, the initial part of this signal was enlarged to show how the time of flight was determined for interfering wave packets.

**Figure 9:**
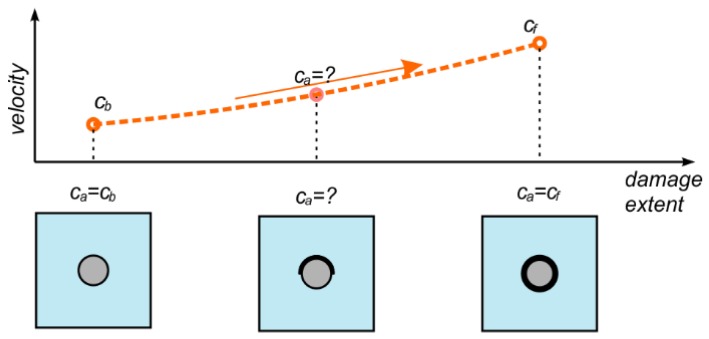
Changes in an average wave velocity depending on debonding size.

It can be seen that the travel time of reflection from the end differs for varying extent of circumferential deboning. For larger debonding extent, the first reflection was registered earlier. Characteristic waveforms were indicated by a straight line passing through wave packets. The possibility of tracing a straight line through marked wave packets shows that there can exist a linear relationship between time of flight (ToF) and monitored variable (debonding size). The explanation for the decrease of registration time with extent of circumferential damage lies in the difference between velocities of the fastest modes in free rod and in reinforced concrete beam. The dispersion solution shows that for a frequency of 60 kHz in free rod only one longitudinal mode can propagate, while in concrete beam several wave modes can be excited. The mode with the highest velocity is registered in signal as a first. For excitation frequency of 60 kHz the velocity of the fastest longitudinal mode in free rod obtained theoretically was *c_f_* = 4896.8 m/s (see Figure 3). The velocity of the fastest mode in reinforced concrete beam was about *c_b_* = 2760.3 m/s. For undamaged beam, the first mode traveled with velocity *c_b_*, but as a result of gradual detachment of the rod the wave velocity tended to the velocity *c_f_* in the free, uncovered rod (Figure 9).

Assuming that the average travel time *t_a_* is linearly dependent on the damage size, the following formula can be defined:

(12) $$t_{a}=t_{b}-\frac{d_{e}}{360{}^{\circ}}\cdot \left(t_{b}-t_{f}\right),$$

where *d_e_* is the circumferential damage extent given in degrees, *t_b_* is travel time for undamaged beam:

(13) $$t_{b}=\frac{L_{a}}{c_{b}},$$

and *L_a_* is the total length of the concrete beam. The *t_f_* is a travel time for uncovered, free rod:

(14) $$t_{f}=\frac{L_{a}}{c_{f}}.$$

the average velocity *c_a_* can be then described by a function:

(15) $$c_{a}=\frac{L_{a}}{t_{a}}=\frac{L_{a}}{\frac{L_{a}}{c_{b}}-\frac{d_{e}}{360{}^{\circ}}\cdot \left(\frac{L_{a}}{c_{b}}-\frac{L_{a}}{c_{f}}\right)}=\frac{1}{\frac{1}{c_{b}}-\frac{d_{e}}{360{}^{\circ}}\cdot \left(\frac{c_{f}-c_{b}}{c_{b}c_{f}}\right)}=\frac{360{}^{\circ}\cdot c_{b}c_{f}}{360{}^{\circ}\cdot c_{f}-d_{e}(c_{f}-c_{b})}.$$

The function described by Equation (12) is linear function with a slope:

(16) $$-\frac{1}{360{}^{\circ}}\cdot \left(t_{b}-t_{f}\right)$$

In can be seen that several different cases can take place: when *t_b_* is higher than *t_f_*, the slope is negative and damage development would involve decrease of *t_a_*; when *t_b_* is lower than *t_f_*, then travel time *t_a_* would increase with damage size and when *t_f_* and *t_b_* are equal, then debonding size has no influence on the average travel time. It is noteworthy that the higher the slope, the greater impact of debonding on travel time and in consequence greater change in average wave velocity would be observed. The relationship in Equation (15) indicates that the influence of circumferential debonding size on wave velocity is completely different than influence of debonding length described in [28]. The correctness of the proposed relationship in Equation (12) was verified using experimental results. On the basis of registered signals, times of flight were determined for variable damage extent. The travel time was calculated as follows: the envelope of the analyzed signal was calculated using Hilbert transform [40]:

(17) $$\overset{⌢}{u}(t)=\frac{1}{\pi }\int_{-\infty }^{\infty }\frac{u(\tau )}{t-\tau }d\tau$$

where $\overset{⌢}{u}(t)$ is Hilbert transform for function *u*(*τ*) defined for all *t*. Then, the first peak location was found and the time was reduced by half the duration of the input wave packet [41] (in the case of excitation frequency of 60 kHz half the duration of the input packet was equal to 8.333×10^−5^ s). Particular times of flight were summarized in Table 1. Then, average wave velocity in the whole beam *c_a_* was calculated:

(18) $$c_{a}=\frac{L_{a}}{t_{a}}.$$

the length of the beam was assumed here as *L_a_* = 0.476 m and was equal to length of the embedded part of the rod (see Figure 4a). Next, experimental outcomes were compared with theoretical results obtained using Equation (15). One can see, that a high level of compliance between experimentally and theoretically determined wave velocities is observed. The maximum absolute error is equal to 3.9%.

**Figure 10:**
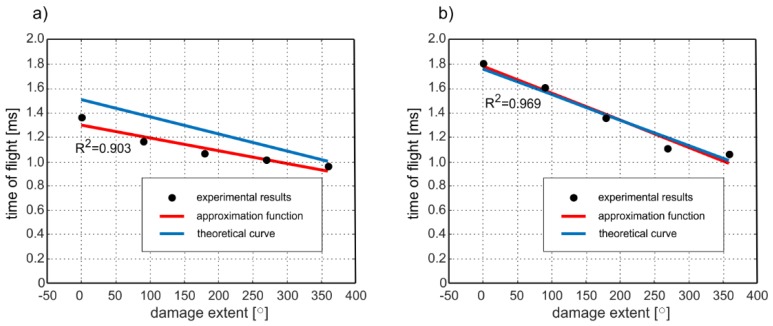
Times of flight of reflections registered at the end of the beams for excitation frequency of (**a**) 40 kHz and (**b**) 50 kHz for variable extent of debonding.

In the next step, the same analysis was conducted for different parameters of excitation. The procedure for determining the time of flight was carried out for excitation frequencies of 40 and 50 kHz. Conducting an experiment and calculations for various frequencies allows verifying the correctness of proposed relationship for different wave velocities *c_f_* and *c_b_*. In Figure 10 the experimentally determined times of flight, which were approximated by a linear function based on regression analysis (least squares method), are presented. The qualitative assessment of the approximation was made using the R-squared ($R^{2}$) correlation coefficient. The correlation coefficients of 0.903 and 0.969 indicate the high strength of the correlation. Next, the experimentally obtained results were compared with theoretical predictions. Blue solid lines represent functions plotted using Equation (12) for theoretically determined times *t_f_* and *t_b_* on the basis of dispersion curves. Despite the fact that the individual theoretical and experimental curves do not coincide perfectly, their slopes are comparable, especially in the case of results obtained for 50 kHz (Figure 9b). The slope of the curve described by Equation (12) is a measure of the impact of damage size on the time of flight (and in consequence average wave velocity). The higher the slope, the greater the change in velocity due to damage development. Comparable slopes of the theoretical and experimental curves indicate that both experimental investigation and theoretical analysis demonstrated a comparable effect of damage presence on propagation velocity.

Despite the fact that for both frequencies, 40 and 50 kHz, a good agreement between theoretical and experimental curve slopes is observed, the higher compliance of results is clearly visible for 50 kHz (Figure 9b). Different compatibility levels for different frequencies could be caused by imperfections that were not included in the theoretical model. Times of flight *t_f_* and *t_b_* were obtained from dispersion curves computed for perfect, isotropic, elastic, homogeneous material. Moreover, the theoretical model assumed that steel rod was placed centrically. In fact, concrete is composite material that consists primarily of mortar and aggregates of different sizes and properties. In addition, the real objects are usually characterized by some deviation from symmetry. Material and geometric imperfections can be the reason for some discrepancies between theoretically and experimentally determined velocities [42].

## 4. Numerical Analysis

Numerical investigation of guided wave propagation in a concrete beam were performed using finite element method in commercial programme Abaqus/Explicit environment. Eight-nodes brick finite elements with reduced integration (C3D8R) were used to develop beams models. In order to choose an appropriate element size, the mesh convergence study was performed. Dimensions of every element were the same and were approximately equal to 2 mm × 2 mm × 2 mm. The transient wave propagation problem was solved with the integration time step. Both, element size and length of integration step were assumed on the basis of wavelength and wave velocity. The connection between the rod and the concrete cover was assumed rigid, and modeled as a tie constraint. In case of modeling the debonding between the cover and the steel rod, the connection between steel and concrete was not defined on a certain part. The wave excitation performance was conducted by applying a concentrated force at a node in the middle of the steel rod. The load was applied as a sine function modulated by the Hanning window. 

Materials were assumed as elastic and isotropic. Material parameters and geometry of the model as well as parameters of excitation load (number of cycles of sine) were introduced in ABAQUS on the basis of experimental data. The results are presented for excitation frequency of 60 kHz.

**Figure 11:**
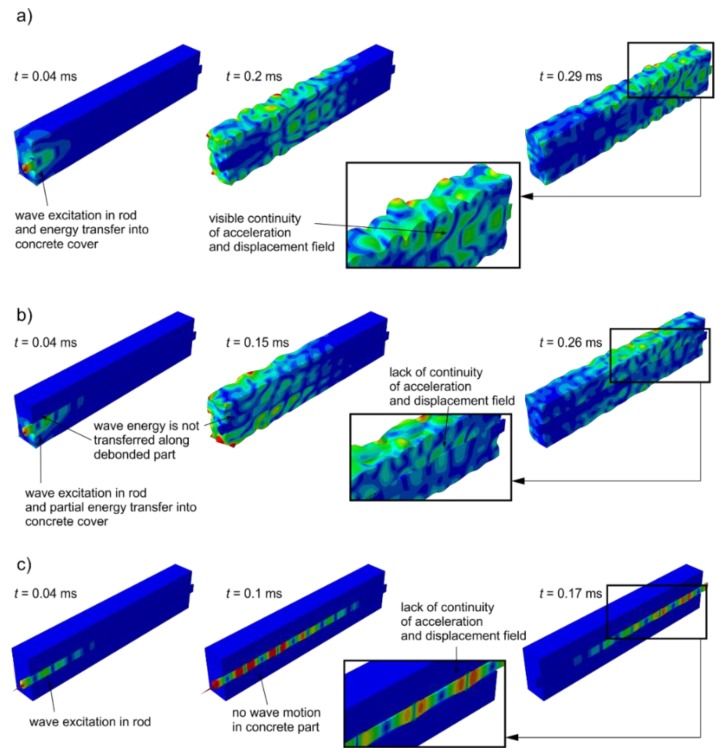
Numerical results of wave propagation in (**a**) undamaged beam and beams with circumferential deboning of extent of (**b**) de = 180° (beam #C) and (**c**) de = 360° (beam #E) for excitation frequency 60 kHz.

To compare the wave propagation phenomena in undamaged and damaged beam, a number of snapshots illustrating the magnitude of acceleration and the deformation of the beam caused by wave motion in selected time instances were set in Figure 11. In the first row (Figure 11a) results of numerical analysis of wave propagation in undamaged beam #A are presented. The second row contains results obtained for beam #C (see Figure 2, *d_e_* = 180°) and third row contained snapshots for beam #E with totally debonded rod (*d_e_* = 360°). Then, wave excitation in the rod perfectly bonded with the concrete cover part of wave energy is transferred into surrounding medium, while part propagates in steel rod (Figure 11a). All the time the wave propagated in the beam, the continuity of acceleration and displacement fields caused by wave motion were visible (Figure 11a, *t* = 0.2 ms and *t* = 0.29 ms). 

For beams with partial circumferential debonding, the differences in wave propagation phenomenon were visible from the very beginning, after wave excitation (Figure 11b). When partial debonding was introduced to the model, wave leaks occurred into surrounding medium only along the part, where rod cooperates with the concrete cover. Unlike in the previous case, the lack of continuity of displacement and acceleration fields was observed. When the rod was totally debonded (Figure 11c), the wave was excited and propagated only along the steel insert. In the concrete part, any wave motion was visible. Except for the differences in acceleration and displacements fields continuity, numerical simulations showed that the wave travels with various velocities depending on the quality of the steel–concrete adhesive connection. The wave reflections from the end of the beams given in the last snapshots were observed at different times for various damage extents. The wave propagates the slowest in specimens with a perfectly bonded rod (Figure 11a), while the fastest propagates in the separated rod (Figure 11c). Numerical simulations indicate that an average velocity increases for increasing damage extent, which is consistent with the results presented in previous paragraph concerning experimental investigation.

**Figure 12:**
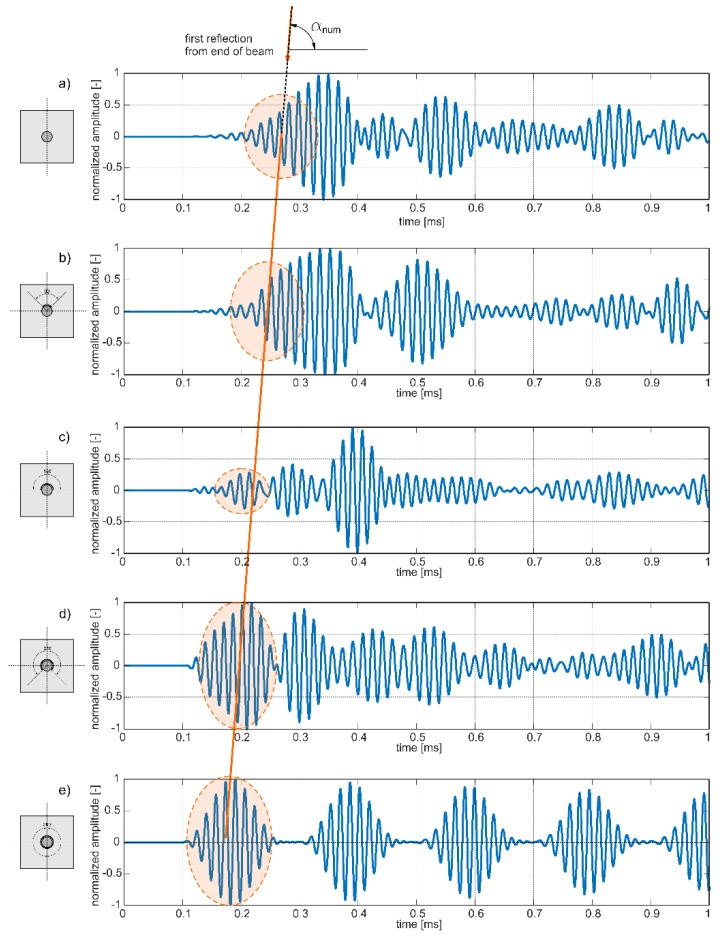
Numerical wave propagation signals registered at the end of the beam with variable circumferential debonding extent: (**a**) undamaged beam and beam with debonding extent of (**b**) 90°, (**c**) 180°, (**d**) 270°, and (**e**) 360° for excitation frequency 60 kHz.

In addition, Figure 12 presents a set of numerical signals registered at the ends of the five beam models #A–#E. The straight line connects wave packets, which first reached the end of the specimen and indicate a linear relationship between time of flight and damage size. Despite the fact that, the character of experimental and numerical signals differ slightly, the slopes of straight lines denoted as α_exp_ and α_num_ are identical in both cases (see Figure 7 and Figure 12). It can be seen that also in the case of numerical results an interference of particular wave packets was observable (Figure 12a,b). As previously, only the initial part of the first wave packet was highlighted and taken into account when time of flight was calculated.

## 5. Discussion of Aspects of Practical Application

### 5.1. The Algorithm of Debonding Detection

Equation (15) can be easily reformulated:

(19) $$d_{e}=\frac{360{}^{\circ}c_{f}\left(c_{a}-c_{b}\right)}{c_{a}\left(c_{f}-c_{b}\right)}.$$

On the basis of above reformulation it can be concluded that the debonding extent can be estimated using the velocities of the fastest modes, which can be determined theoretically and average wave velocity *c_a_*, obtained experimentally. The proposed procedure for debonding size estimation is as follows:

1. On the basis of the cross-section geometry and material parameters of the concrete and steel, trace the dispersion curves.
2. Determine the theoretical velocities of the fastest modes *c_f_* and *c_b_* for frequency, which is used in experimental tests.
3. Determine experimentally the average velocity *c_a_* of the first reflection.
4. Use the Equation (16) to calculate the debonding extent.

The proposed algorithm does not require any reference data collected for the undamaged structure, and can be applied in diagnostics of embedded bars embedded in various types of concrete, as long as it can be considered as elastic and isotropic material.

The main drawback of the described algorithm is its sensitivity to inaccuracies in estimation of velocities/times of flight: the slight error in velocity estimation will result in significant over- or under-estimation of the debonding extent. From a practical point of view, the inaccuracies can be minimized by repeating the measurement with various excitation frequencies and various *c_f_* and *c_b_*. Collecting data for more than one frequency allows for better assessment of the damage size.

The developed algorithm requires experimental verification with considering a various ways of wave excitation. For the existing, actual structures it is usually impossible to attach the sensor and actuator at both ends of the embedded rod, and the most practical solution would be to excite wave by placing the sensor on the side surface, perpendicularly to the beam axis. Perpendicular attachment of the actuator triggers flexural modes in the concrete cross-section, what should be taken into account when determining *c_f_* and *c_b_*. Furthermore, it is necessary to consider how the propagation phenomenon changes when wave is excited eccentrically, not directly in the rod. Moreover, the diagnostics of actual reinforced concrete structures of a significant size involves the necessity of excitation of signals characterized by much higher energy.

### 5.2. Detection of Multiple Debondings

**Figure 13:**
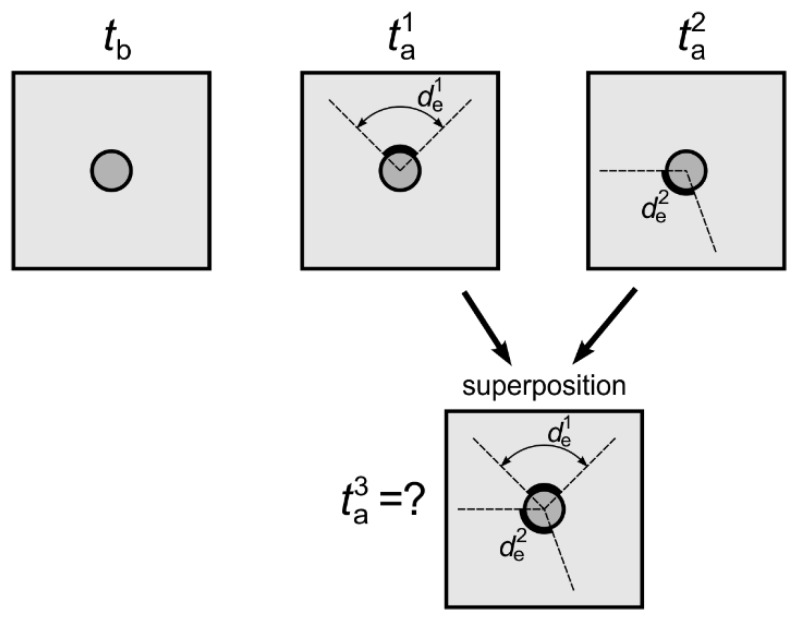
Scheme of specimen with multiple debondings.

Let’s consider the case of two debondings occurring along the whole rod with different circumferential extent $d_{e}^{1}$ and $d_{e}^{2}$ (Figure 13). To analyze the total influence of both damages on wave velocity, in the first step the impact of individual debondings on time of flight is considered singly. 

In the case of fully bonded, healthy rod the time of flight of the fastest mode is equal to *t_b_*. To calculate the times of flight for individual debondings, the Equation (12) can be used:

(20) $$t_{a}^{1}=t_{b}-\frac{d_{e}^{1}}{360{}^{\circ}}\cdot \left(t_{b}-t_{f}\right),$$

(21) $$t_{a}^{2}=t_{b}-\frac{d_{e}^{2}}{360{}^{\circ}}\cdot \left(t_{b}-t_{f}\right),$$

The development of particular debondings $d_{e}^{1}$ and $d_{e}^{2}$ causes the shortening the times of flight by $\Delta t_{a}^{1}$, $\Delta t_{a}^{2}$, respectively:

(22) $$\Delta t_{a}^{1}=t_{b}-t_{a}^{1}=t_{b}-\left(t_{b}-\frac{d_{e}^{1}}{360{}^{\circ}}\cdot \left(t_{b}-t_{f}\right)\right)=\frac{d_{e}^{1}}{360{}^{\circ}}\cdot \left(t_{b}-t_{f}\right),$$

(23) $$\Delta t_{a}^{2}=t_{b}-t_{a}^{2}=t_{b}-\left(t_{b}-\frac{d_{e}^{2}}{360{}^{\circ}}\cdot \left(t_{b}-t_{f}\right)\right)=\frac{d_{e}^{2}}{360{}^{\circ}}\cdot \left(t_{b}-t_{f}\right),$$

Then, the development of two debondings entails the shortening the time of flight *t_b_* by $\Delta t_{a}^{1}+\Delta t_{a}^{2}$

(24) $$\begin{array}{c} t_{a}^{3}=t_{b}-\Delta t_{a}^{1}-\Delta t_{a}^{2}=t_{b}-\frac{d_{e}^{1}}{360{}^{\circ}}\cdot \left(t_{b}-t_{f}\right)-\frac{d_{e}^{2}}{360{}^{\circ}}\cdot \left(t_{b}-t_{f}\right)\\ =t_{b}-\frac{\left(d_{e}^{1}+d_{e}^{2}\right)}{360{}^{\circ}}\cdot \left(t_{b}-t_{f}\right)=t_{b}-\frac{d_{e}^{t}}{360{}^{\circ}}\cdot \left(t_{b}-t_{f}\right), \end{array}$$

where $d_{e}^{t}$ is the total extent of the debonding. Using the superposition, it can be concluded that the time of flight and wave velocity depend on the total circumferential extent of the damage, regardless of whether single or multiple debondings occur.

### 5.3. Influence of Debonding on Wave Amplitude

Based on the numerical results presented in the form of snapshots of propagating waves (Figure 11), it is clearly visible that the damage size has a great influence on wave energy leakage. When waves are excited in fully bonded rods, wave motion energy can be easily transferred into surrounding medium along the whole rod surface adhesively connected with concrete cover.

**Figure 14:**
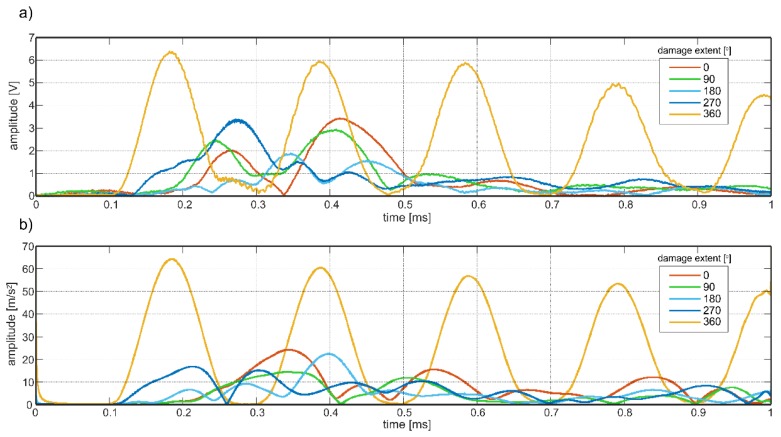
Envelopes of signals obtained (**a**) experimentally and (**b**) numerically for varying extent of debonding.

To present the influence of damage size on wave amplitude, experimental and numerical results in the form of envelopes of unnormalized registered at the ends of the particular beam models were collected in Figure 14. Despite the fact that, there is no clear relationship between amplitude and damage extent, the significant difference between amplitude for totally bonded rod and partially damaged specimens is visible in both cases. The significant amplitude increase can be effectively used in detecting totally debonded rods, especially in continuous monitoring systems, which take advantage of permanently attached sensors.

## 6. Conclusions and Future Plans

The present study concerns theoretical, experimental, and numerical investigations of guided wave propagation in reinforced concrete beams with artificially introduced miniscule debonding (90 μm). A number of reinforced concrete beams with the same length of debonding but varying in size of circumferential direction were investigated. Five damage scenarios were taken into consideration: the debonding extent was 0, 90, 180, 270, or 360 degrees, respectively.

The main aim of the research was analyzing if circumferential debonding has an impact on average wave propagation velocity. The experimental investigations consisted of wave excitation at the start and signal receiving at the end of the beam with the use of piezoelectric sensors. Based on the captured time–domain signals, the time of flight and next wave velocity were determined for varying damage extents. Analysis of the results obtained showed that wave velocity in beams increases with increasing debonding size. It was shown that the relationship between time of flight and debonding extent can be approximated by a linear function and then, the relationship between average wave velocity and debonding size has been derived. The expression describing average wave velocity was developed based on dispersion curves for rectangular reinforced and circular cross-sections of steel rods. The formula was derived with the assumption that velocity of the fastest mode in undamaged beam *c_b_* tends to the velocity *c_f_* in the free, uncovered waveguide because of gradual detachment of the rod. The proposed linear function relating ToF and debonding size is characterized by initial value equal to *t_b_*, which the arrival time of the fastest mode in the undamaged beam and the slope, which is proportional to the difference between times of flight the fastest modes for particular parts (*t_b_* – *t_f_*). Depending on the slope of the function, three cases can be distinguished: *t_b_* is higher than *t_f_*, the slope is negative and damage development would involve decrease of time of flight of the first reflection *t_a_*; when *t_b_* is lower than *t_f_*, then debonding development would increase the travel time *t_a_* and when *t_f_* and *t_b_* are equal, the slope is zero and then debonding size has no influence on average travel time. 

The slope of the function can be interpreted as sensitivity. The greater the slope is, the more sensitive the function is. Knowledge about the function and its slope allow predicting which frequencies are the most sensitive to debonding development. When the slope is large, even a small change in debonding extent is related to significant change in average velocity. The obtained results suggest that it is more efficient to choose the frequencies for which the difference *t_b_* − *t_f_* (or *c_f_* − *c_b_*) is as high as possible.

In this study, signals were collected for three different excitations frequencies: 40, 50, and 60 kHz. On the basis of dispersion curves traced for concrete beam and free, steel waveguide it can be concluded that the biggest difference between velocities *c_f_* and *c_b_* for frequency 40 kHz was smaller than for 50 and 60 kHz. For this reason the frequency 40 kHz was characterized by the lowest sensitivity to debonding detection and for this frequency theoretically predicted results were the most deviated from experimental results.

The discrepancies between theoretical and experimental outcomes may also result from imperfections that were not included in the theoretical model. Material and geometric imperfections, eccentricity of excitation, inaccuracy in determining the material parameters or in calculation of time of flight could be the reason for the difference between theoretical and experimental outcomes.

The last stage of investigation concerned numerical calculations of wave propagation in damaged beams. The results given in the form of snapshots for selected time instants identified changes in wave propagation phenomenon for varying damage extent. The main difference was in the amount of wave energy, which leaks into surrounding medium. Energy can be transferred into concrete block only along the part, where rod cooperates with the concrete cover. When the debonding was introduced into the beam model, at the border of media the lack of continuity of displacement and acceleration fields was observed. Meanwhile, the continuity of displacement fields is one basic assumption of the theoretical model describing wave propagation in multilayered specimens. When this assumption is not met, wave velocity predicted based on theory differs from the velocity actually registered in a model. The differences in wave velocities for varying damage size was visible in the performed visualizations: similarly to the experiment velocity increased with circumferential debonding size. In addition, the correctness of the numerical model has been confirmed by comparison numerically and experimentally registered signals for excitation frequency of 60 kHz. In both cases, the slopes of lines connecting reflections from the end of the beams were the same. Identifying the slopes proved that the influence of damage development on average wave velocity obtained numerically and experimentally was identical.

The presented investigation indicates that the influence of size of partial circumferential debonding is completely different from the influence of debonding length described in previous papers. In both cases, the time of flight depends on velocities of the fastest modes and in both cases the slope of the curves is the measure of sensitivity of particular frequencies to debonding detection. However, derived relationships differ and cannot be used interchangeably. In the future, the knowledge about the impact of both, debonding length, and circumferential extent can be used to formulate relationships between average velocity and total debonding area, which would allow for nondestructive evaluation of load capacity of the entire reinforced concrete structure.

## Figures and Tables

**Table 1:** Identifying the time of flight on the basis of signals registered at the end of the concrete beam with variable circumferential debonding extent.

Debonding Extent [°]	Registration at the End of the Beam [ms]		Wave Velocity [m/s]		Absolute Error [%]
	Time Od Flight of the First Peak	ToF (*t_a_*) Reduced by a Half of Input Packet	Calculated on the Basis of ToF (*t_a_*)	Calculated on the Basis of Equation (7)	
0	0.260	0.177	2694.3	2760.3	2.4
90	0.240	0.157	3038.3	3098.3	1.9
180	0.216	0.133	3587.9	3530.5	1.6
270	0.195	0.112	4262.7	4102.9	3.9
360	0.182	0.099	4824.3	4896.8	1.5
